# Nonablative Fractional 1927‐nm Laser for Periorbital Rejuvenation: A Prospective, Double‐Arm, Open‐Label Trial

**DOI:** 10.1111/jocd.70274

**Published:** 2025-06-19

**Authors:** Chang‐Ming Huang, Yi‐Shuan Sheen, Yi‐Hua Liao

**Affiliations:** ^1^ Department of Dermatology National Taiwan University Hospital Taipei Taiwan; ^2^ Department of Dermatology National Taiwan University Hospital Hsin‐Chu Branch Hsinchu Taiwan; ^3^ Department of Dermatology, College of Medicine National Taiwan University Taipei Taiwan

**Keywords:** 1927‐nm, diode laser, fractional, nonablative, periorbital rejuvenation

## Abstract

**Background:**

The 1927‐nm nonablative fractional laser is widely used for skin rejuvenation and hyperpigmentation. However, its efficacy and safety for periorbital rejuvenation remain uncertain.

**Aims:**

To assess the effects of the 1927‐nm nonablative fractional diode laser on periorbital rejuvenation.

**Patients/Methods:**

Twenty participants were randomized into two groups and received three treatments at 2‐week (Group A) or 4‐week (Group B) intervals. Clinical efficacy was assessed using ANTERA 3D, blinded investigator‐rated scores, and subject self‐assessments.

**Results:**

Both group A and group B exhibited statistically significant improvements in L* (color measurement which indicates lightness) and pigmentation score at the end‐of‐study visit compared to baseline, with group B also achieving statistically significant enhancement in the pores' index and wrinkles' score. Group B showed greater improvement in wrinkles' score compared to group A at the end‐of‐study visit. The median of Global Aesthetic Improvement Scale assessed by both the physicians and subjects showed improvement, ranging from “improved” to “much improved” at the end‐of‐study visit. Transient side effects including tenderness, erythema, swelling, and scaling were observed, with the majority resolving within 7 days.

**Conclusions:**

The 1927‐nm diode laser is an effective and safe option for periorbital rejuvenation. The 4‐week interval treatment appears to be the preferable option due to its superior improvement in the wrinkles' score at the end of the study. The positive changes in skin brightness and the reduction of brown spots represented additional benefits, setting it apart as a notable alternative to other ablative and nonablative fractional lasers.

## 1 Introduction

The periorbital region plays a unique role in facial aging. It is often considered the most expressive and susceptible area of the face. The skin on the eyelids is the thinnest on the body and lacks subcutaneous fat [1]. The thickness of the eyelid skin (including the epidermis and dermis) ranges from 799.16 to 1265.67 μm [2]. Due to frequent muscle contractions, the eyelid skin is particularly prone to wrinkling and visible signs of aging.

Various methods are currently utilized for periorbital anti‐aging, including topical retinoids [3], radiofrequency [4], intense pulsed light [5], chemical peels [6], botulinum toxin [7], platelet‐rich plasma (PRP) [8], and both non‐ablative and ablative laser resurfacing procedures [4, 9]. Full surface ablative resurfacing procedures have proven effective in addressing periorbital wrinkles and laxity [10, 11]. However, the ablation of the epidermis can result in a recovery period lasting 1–2 weeks. Posttreatment erythema and hyperpigmentation are common, especially in individuals with darker skin types. The application of periorbital laser resurfacing carries a potential risk of developing lower lid ectropion [11]. Challenges persist in the rejuvenation of the periorbital area.

In 2012, the FDA granted approval for the 1927 nm wavelength handpiece, a nonablative fractional diode laser [12]. Its primary purpose is skin rejuvenation, focusing on early signs of facial aging, sun damage, improving skin tone and texture, and reducing pore size [12, 13, 14, 15, 16]. However, its efficacy as well as the treatment protocol in periorbital rejuvenation remain uncertain, along with potential risks and adverse effects. Therefore, our objective is to evaluate the impact of periorbital rejuvenation using the 1927 nm wavelength nonablative fractional diode laser system.

## 2 Materials and Methods

### 2.1 Participants

The inclusion criteria encompassed individuals aged between 30 and 65 years old with no significant inflammatory lesions on the face. Exclusion criteria comprised participants who had undergone active facial treatments including energy‐based devices and injectables in the last 6 months, those with chronic skin conditions such as atopic dermatitis, psoriasis, chronic urticaria, vitiligo, rosacea, or keloid, pregnant or breastfeeding individuals, those suffering from acute illnesses or infections requiring treatment within 14 days before entering the study, individuals with serious illnesses (such as heart, lung, brain, or liver disease) within the previous 3 months, those allergic to lidocaine or prilocaine used in topical anesthetic cream, and those who had used any topical medication on the face within 30 days before the trial. Twenty subjects were recruited for the study, and none of them withdrew. Informed consent was obtained from all participants.

### 2.2 Study Design

**FIGURE 1:**
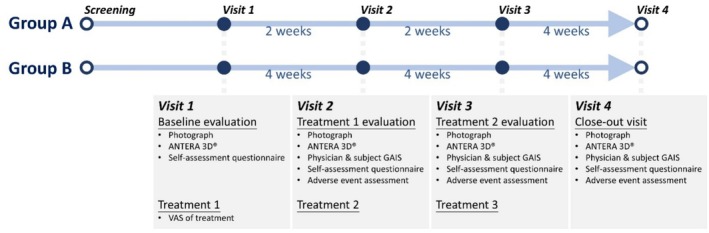
Study design. The study design is illustrated in the figure. Participants were randomly assigned to two groups. The treatment and evaluation at each visit are detailed in the figure.

This was a prospective randomized clinical trial aimed at evaluating the efficacy and optimal treatment interval for periorbital rejuvenation using the 1927‐nm diode system [Clear+Brilliant diode laser system; Solta Medical]. The research project focused on exploring the treatment design, therapeutic effects, and safety of the 1927 nm laser for skin rejuvenation around the eyes. Conducted as an open‐label, single‐center clinical trial, participants were randomly assigned to two treatment groups, namely group A (with a treatment interval of 2 weeks) or group B (with a treatment interval of 4 weeks). The study design was illustrated in Figure 1. This study received approval from the Research Ethics Committee of NTUH (202207219DSC) and was registered on ClinicalTrials.gov (NCT05811026).

### 2.3 Randomization and Procedures

Subjects were randomly assigned to either group A (with a 2‐week treatment interval between treatments) or group B (with a 4‐week interval between treatments). Each group comprised 10 subjects. In group A, the intervals between the first, second, and third treatments were 2 weeks, with the end‐of‐study visit occurring 4 weeks after the third treatment. In group B, the intervals between the first, second, and third treatments were 4 weeks, and the end‐of‐study visit took place 4 weeks after the third treatment.

### 2.4 Investigated Laser System and Procedures

The laser system under investigation is the Clear+Brilliant diode laser system equipped with the 1927 nm wavelength handpiece, produced by Solta Medical Inc., Hayward, CA. During each treatment session, a 1927 nm diode laser with 5 mJ energy, a treatment spot of 140 μm, a treatment depth of 170 μm, and a treatment coverage of approximately 5% per energy level was used. This was administered over four rounds of treatment utilizing the patented Intelligent Optical Tracking System (IOTs). The treated area extended from the lower margin of the eyebrow to the nasal ala and from the nasal bridge to 1 cm lateral to the lateral canthus on each side of the face.

### 2.5 Efficacy Assessment

Efficacy is evaluated through the utilization of ANTERA 3D (Miravex Limited, Dublin, Ireland) and the Global Aesthetic Improvement Scale Assessment (GAIS) conducted by both physicians and subjects.

ANTERA 3D, a specialized medical skin imaging equipment, analyzes shadow and light reflectance to generate a three‐dimensional color image of the skin surface. It facilitates a swift, straightforward, and precise analysis and measurement of wrinkles, pores, texture, pigmentation, redness, and color [17]. In this study, the assessed ANTERA 3D parameters include L* values (color measurement which indicates lightness), texture score, pigmentation score, pores' index and wrinkles' score. The primary outcome is the change from baseline in the texture score at visit 4 (end‐of‐study visit). The secondary outcome encompassed the change from baseline in L* (color measurement), texture score, pigmentation score, and pores' index at visit 2 to visit 4.

The GAIS, a 5‐point scale ranging from 1 to 5 (1 = very much improved, 2 = much improved, 3 = improved, 4 = no change, and 5 = worse), was also employed. Changes from baseline in GAIS at visit 2 to visit 4 were recorded. Physician GAIS was independently assessed by three board‐certified dermatologists with reference to photography records, while the subject GAIS was self‐rated by the individual.

### 2.6 Self‐Assessment Questionnaire

Every participant in the study filled out a self‐assessment questionnaire regarding the response to each treatment. Each question was rated on a scale of five: “very satisfied,” “satisfied,” “neutral,” “unsatisfied,” or “very unsatisfied.” The questions were initially presented in Chinese and were translated into English for this article. The specific questions are provided in the table.

### 2.7 Pain and Adverse Event Assessment

The visual analogue scale was applied for rating the pain immediately after the first laser treatment. Also, each subject rated the presence of erythema, swelling, itchiness, tenderness, and scaling using a 3‐point scale (0 = none, 1 = mild, 2 = moderate, and 3 = severe) after each treatment, which had records of a total 60 person‐times of treatment (Table 5).

### 2.8 Statistical Analysis

The Wilcoxon signed‐rank test was used to compare ANTERA 3D parameters and each item in the self‐assessment questionnaire at each visit to baseline. The Mann–Whitney U test was employed to assess differences in the improvement of ANTERA 3D parameters and GAIS between the two groups. Statistical analysis was performed using SAS 9.4 (Cary, North Carolina, USA). All tests were two‐sided. *p* values of < 0.05 were regarded as statistically significant.

## 3 Results

### 3.1 Participants

A total of 20 participants were screened and enrolled in this study, with 10 participants in each group. The enrolled participants included one man and nine women in each group, aged between 39 and 61 years, with skin phototypes III‐IV. In group A, the median age was 53 years, and in group B, the median age was 45 years (*p* = 0.019; Table 1).

**TABLE 1:** Baseline characteristics.

Parameter (median)	A	B	*p*
Age, years	53	45	0.019^a^
Sex, no. (%)			
Female	9 (90)	9 (90)	1.000
Male	1 (10)	1 (10)	
Texture score	32	34.25	1.000
L*	58.09	59.45	0.821
Pigmentation score	54.5	50.25	0.384
Pores' index	0.98	1.36	0.226
Wrinkles' score	82.75	106.5	0.280

*Note:* For continuous variables, data are presented as medians.

^a^ *p* ≤ 0.05. The *p* value was calculated using a two‐tailed Mann–Whitney *U* test. For categorical variables, data are presented as numbers and percentages. The *p* value for categorical variables was calculated using a two‐tailed chi‐squared test.

### 3.2 Analyses of Clinical Outcomes

#### 3.2.1 Biophysical Measurements

**FIGURE 2:**
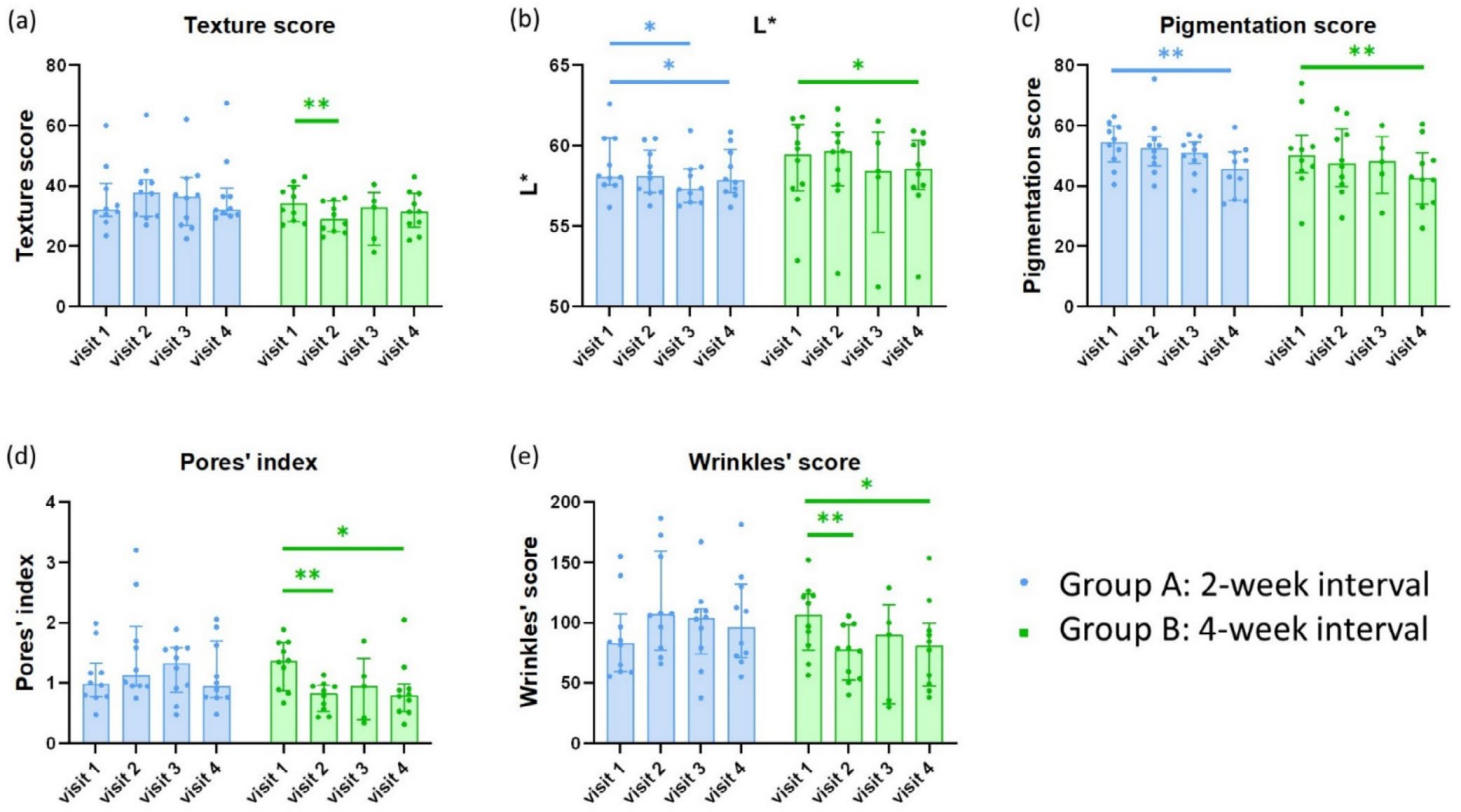
Comparison of skin features across visits. The bar chart illustrates the results of skin topographic measurements by ANTERA 3D for each group and visit. The assessed ANTERA 3D parameters include L* values (color measurement which indicates lightness), texture score, pigmentation score, pores' index and wrinkles' score. Data are shown as medians with interquartile range. *p ≤ 0.05, **p ≤ 0.01, ***p ≤ 0.001. *p* value was calculated using a two‐tailed Wilcoxon signed‐rank test.

**FIGURE 3:**
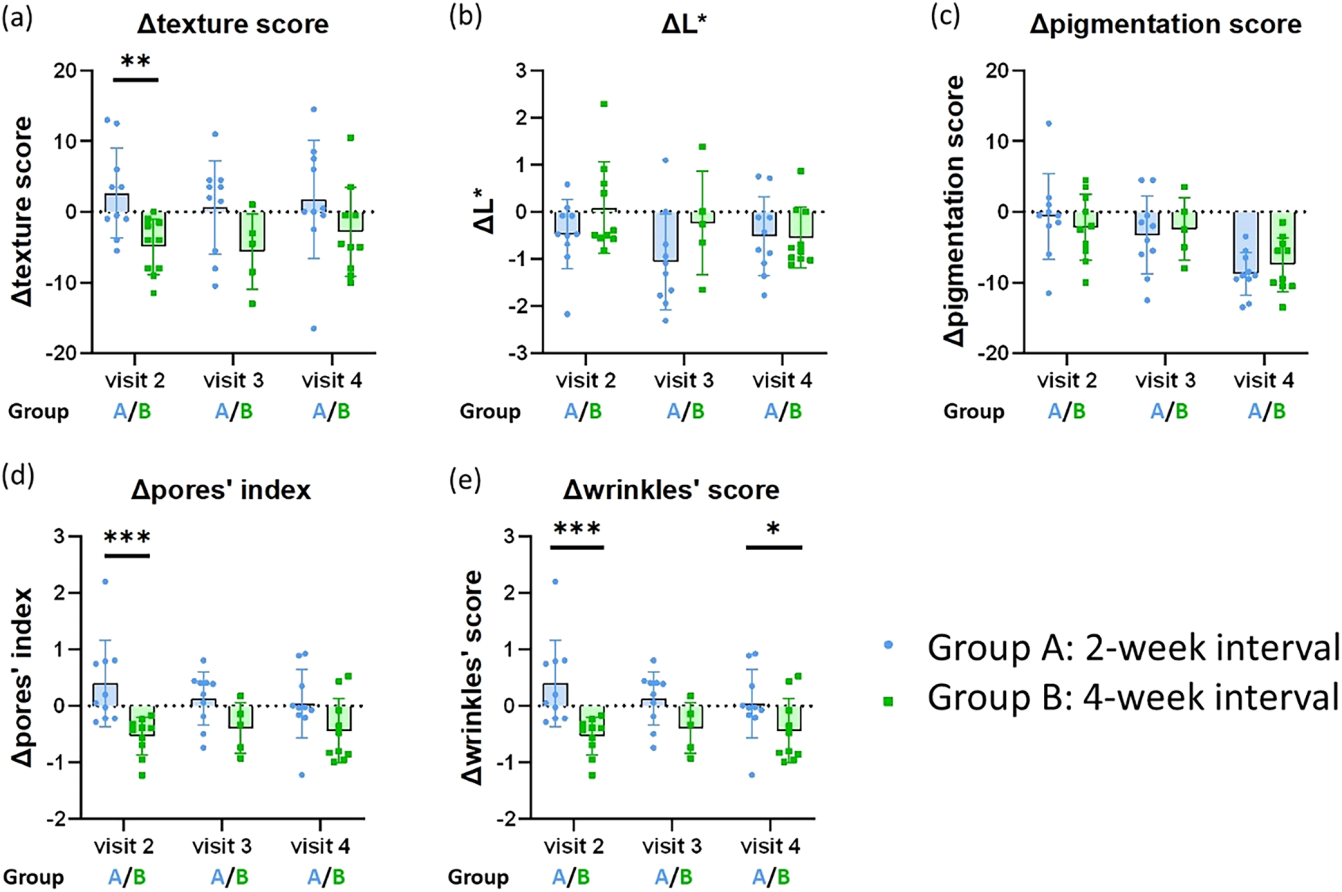
Comparison of skin features changes between groups. The bar chart compares the change from baseline in ANTERA 3D parameters at each visit between groups. The assessed ANTERA 3D parameters include L* values (color measurement which indicates lightness), texture score, pigmentation score, pores' index and wrinkles' score. Data are shown as medians. *p ≤ 0.05, **p ≤ 0.01, ***p ≤ 0.001. *p* value was calculated using a two‐tailed Mann–Whitney *U* test.

At baseline, there were no differences in texture score, L*, pigmentation score, pores' index, or wrinkles' score (Table 1). The study did not achieve the primary outcome, as the change from baseline in texture score at visit 4 (end‐of‐study visit) was not significant in either group A or group B. In group A, the L* parameter showed a statistically significant improvement at both visit 3 and visit 4 compared to the baseline in visit 1. The pigmentation score also improved at visit 4 compared to the baseline in visit 1. In group B, both the L* parameter and pigmentation score exhibited statistically significant improvement at visit 4 compared to the baseline in visit 1. The texture score improved at visit 2 compared to the baseline in visit 1, and the pores' index and wrinkles' score showed improvement at both visit 2 and visit 4 compared to the baseline in visit 1 (Figure 2, Table 2). When comparing group A and group B, statistically significant differences were observed in the improvement of wrinkles' scores at visit 4, with group B showing better results. Statistically significant differences were also noted at visit 2 in the improvement of texture score, pores' index, and wrinkles' score, all favoring group B. No other statistical differences between the two groups were noted among the remaining parameters (Figure 3, Table 3). Specifically noted, ANTERA 3D was non‐functional due to technical issues during the third visit for 5 subjects in group B, and this data were excluded from the analysis.

**TABLE 2:** Comparison of skin features across visits.

Group	Parameter (median)	Visit 1	Visit 2	*p* ^a^	Visit 3	*p* ^b^	Visit 4	*p* ^c^
A	Texture score	32	37.75	0.4082	36.5	0.7422	32.25	0.4844
	L*	58.09	58.13	0.084	57.32	0.0273*	57.76	0.0195*
	Pigmentation score	54.5	52.5	0.5859	51	0.1016	45.5	0.002*
	Pores' index	0.98	1.12	0.2754	1.33	0.4316	0.95	0.8457
	Wrinkles' score	82.75	106.75	0.0547	103.75	0.375	91.25	0.375
B	Texture score	34.25	29	0.0039*	33	0.125	31.5	0.1504
	L*	59.45	59.64	1	58.43	0.625	58.4	0.0488*
	Pigmentation score	50.25	47.5	0.1992	48	0.375	42.5	0.002*
	Pores' index	1.36	0.83	0.002*	0.95	0.1875	0.8	0.0488*
	Wrinkles' score	106.5	77.5	0.002*	90	0.125	70	0.0391*

*Note:* The assessed ANTERA 3D parameters include L* values (color measurement which indicates lightness), texture score, pigmentation score, pores' index, and wrinkles' score. Data are shown as medians.

^a^ The *p*‐value obtained from the comparison between visit 1 and visit 2.

^b^ The *p*‐value obtained from the comparison between visit 1 and visit 3.

^c^ The *p*‐value obtained from the comparison between visit 1 and visit 4.

* *p* ≤ 0.05. *p* value was calculated using a two‐tailed Wilcoxon signed‐rank test.

**TABLE 3:** Comparison of skin feature changes between groups.

Parameter		A	B	*p*
ΔTexture score	(visit 2–visit1)	1.5	−4	0.0088*
	(visit 3–visit1)	2.75	−4.5	0.0752
	(visit 4–visit1)	0.25	−4.75	0.0750
ΔL*	(visit 2–visit1)	−0.47	−0.44	0.4274
	(visit 3–visit1)	−1.2	−0.26	0.0982
	(visit 4–visit1)	−0.98	−0.81	0.4727
ΔPigmentation score	(visit 2–visit1)	−0.5	−2.25	0.6497
	(visit 3–visit1)	−3	−2.5	0.8541
	(visit 4–visit1)	−9	−7.5	0.7326
ΔPores' index	(visit 2–visit1)	0.13	−0.41	0.0006*
	(visit 3–visit1)	0.3	−0.33	0.0758
	(visit 4–visit1)	−0.03	−0.64	0.0757
ΔWrinkles' score	(visit 2–visit1)	11	−23.5	0.0002*
	(visit 3–visit1)	20.25	−21.5	0.066
	(visit 4–visit1)	12.25	−25.5	0.0376*

*Note:* The assessed ANTERA 3D parameters include L* values (color measurement which indicates lightness), texture score, pigmentation score, pores' index and wrinkles' score. Data are shown as medians.

* *p* < 0.05. *p* value was calculated using a two‐tailed Mann–Whitney *U* test.

In summary, both L* and pigmentation score reached statistically significant improvement in both group A and group B at visit 4, with group B additionally achieving statistically significant improvement in the pores' index and wrinkles' score at visit 4. At visit 4, only the wrinkles' score showed a statistically significant difference between the two groups, with group B showing better results.

#### 3.2.2 Global Aesthetic Improvement Scale (GAIS)

**FIGURE 4:**
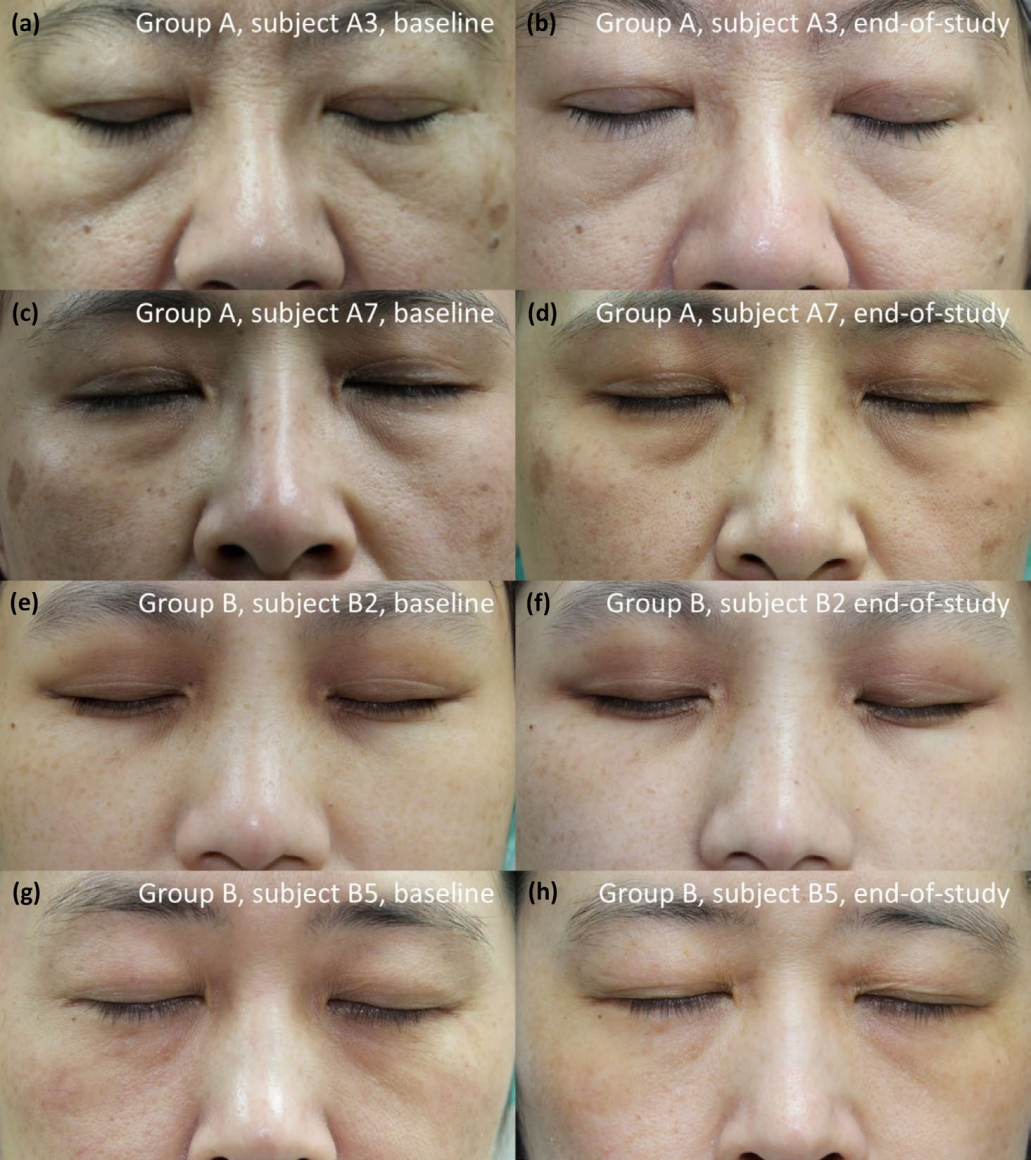
Representative clinical photographs at baseline and end‐of‐study visit from four subjects. Baseline and end‐of‐study visit photographs of four subjects are displayed (a and b) Baseline and end‐of‐study visit photographs of a subject from group A; (c and d) Baseline and end‐of‐study visit photographs of another subject from group A; (e and f) Baseline and end‐of‐study visit photographs of a subject from group B; (g and h) Baseline and end‐of‐study visit photographs of another subject from group B. The improvement in fine wrinkles, skin texture, brown spots, and dark circles can be observed.

The detailed data was shown in Table 4. Both group A and group B showed median grading of 2 (much improved) to 3 (improved) at visit 4 (end‐of‐study visit). Among physician or subject GAIS, only subject GAIS during visit 3 showed a statistically significant difference between group A and B, with better improvement in group B (Table 4). The representative clinical photographs of two subjects from each group are presented in Figure 4.

**TABLE 4:** Comparison of Global Aesthetic Improvement Scale assessment across groups.

Parameter (median)		*A*	*B*	*p*
Physician GAIS	(Visit 2)	4	4	0.6231
	(Visit 3)	3.5	3.67	0.6961
	(Visit 4)	2.83	3	0.3123
Subject GAIS	(Visit 2)	3	3	0.3327
	(Visit 3)	3	2	0.0067*
	(Visit 4)	3	2	0.1009

*Note:* Data are shown as medians.

Abbreviation: GAIS, Global Aesthetic Improvement Scale.

* *p* < 0.05. *p* value was calculated using a two‐tailed Mann–Whitney *U* test.

**TABLE 5:** Adverse events among 60 person‐times of treatment.

Symptoms	None	Mild	Moderate	Severe
Erythema	14 (23.3%)	27 (45.0%)	18 (30.0%)	1 (1.7%)
Swelling	20 (33.3%)	31 (51.7%)	6 (10.0%)	3 (5.0%)
Itchiness	42 (70.0%)	14 (23.3%)	4 (6.7%)	0 (0.0%)
Tenderness	11 (18.3%)	36 (60.0%)	10 (16.7%)	3 (5.0%)
Scaling	27 (45.0%)	26 (43.3%)	6 (10.0%)	1 (1.7%)

#### 3.2.3 Self‐Assessment Questionnaire

Several parameters showed significant improvement in the self‐assessment questionnaire, with detailed information presented in Table S1. Brightness of skin, fairness of skin, translucency of skin, radiance of skin, moisture retention of skin, evenness of skin tone, color of brown spots on the skin, size of brown spots on the skin, overall area of brown spots on the skin, youthfulness of skin appearance, refinement of skin texture, and the feeling that the skin has been revitalized exhibited significant improvement in both group A and group B at visit 4 (Table S1).

#### 3.2.4 Pain and Adverse Event

The pain experienced during the initial laser treatment was rated using a visual analogue scale, yielding a result of 5.5 (median; SD = 2.4). We also documented adverse events following each treatment session of each subject in a total of 60 instances (Tables 5 and S2). The most common symptoms included erythema, swelling, itchiness, tenderness, and scaling. Most of the symptoms were mild. Severe adverse events, such as erythema, swelling, itchiness, tenderness, and scaling, were experienced by no more than 5% of subjects. These discomforts mostly resolved within 7 days. No severe adverse events were noted throughout the treatment course and end‐of‐study visit.

## 4 Discussion

Periorbital rejuvenation is of significant importance because the eyes serve as a central feature in facial appearance and expression. Periorbital aging is often one of the first visible signs of aging, manifesting through the appearance of wrinkles, uneven texture, dryness, and changes in pigmentation. However, handling the periorbital area poses challenges due to its pivotal role in vision and the delicate nature of its skin. It is important to note that excessive treatments could potentially affect vision or cause unwanted scars. Therefore, careful consideration and effective treatments, such as the 1927 nm nonablative fractional diode laser, are critical for achieving safe and aesthetically pleasing results.

Nonablative fractional lasers for facial rejuvenation utilize midinfrared wavelengths (1320 nm to 1927 nm), targeting water as a chromophore while preserving the corneal barrier [12]. Compared to CO_2_ or Er:YAG lasers, nonablative fractional lasers have less water absorption, which results in a more controlled treatment that focuses on the dermis without destroying the overlying epidermis. After treatment, microscopic epidermal necrotic debris (MEND), composed of melanin and thermally damaged keratinocytic debris, formed in the subgranular space within 1 day post‐treatment. By 7 days post‐treatment, the MEND was either intracorneal or shed from the epidermis. During this process, pigmented cells migrate upward from the basement membrane and integrate into the MEND through transepidermal exfoliation. Over time, the MEND is expected to flake off further, contributing to the visible effect of pigment removal and improvement in skin appearance [12]. Histologic assessments supported this timeline of pigment migration and epidermal recovery in a study involving 18 individuals treated on the forearm using the 1927 nm wavelength handpiece of the nonablative fractional lasers system [18]. In our study, both group A and group B exhibited statistically significant improvements in L* and pigmentation at visit 4 compared to baseline, indicating effective skin rejuvenation and pigment reduction.

A noteworthy advantage of nonablative fractional infrared lasers is their targeting of water molecules rather than melanin, enhancing safety profiles for darker skin types. The current clinical applications of the 1927 nm nonablative fractional diode laser encompass photodamage, melasma, and post‐inflammatory hyperpigmentation [13, 14, 15, 16]. In our study, which focused on Asian subjects, both group A and group B exhibited statistically significant improvements in L* and pigmentation at visit 4 compared to baseline. Additionally, group B also achieved statistically significant enhancement in the pores' index and wrinkles' score. While numerous studies have confirmed the efficacy of nonablative fractional lasers in improving skin elasticity and tightening [19, 20], the aspects of skin toning and pigment lightening are often less emphasized in other ablative or non‐ablative fractional lasers for rejuvenation. This makes the 1927 nm diode laser particularly valuable for skin brightening and pigmentation reduction, alongside its benefits for texture and elasticity. The lasers commonly used for periorbital rejuvenation are summarized in Table S3.

It is interesting to note that a 4‐week interval treatment is preferred over a 2‐week interval for periorbital rejuvenation with the 1927 nm diode laser in our study. As indicated by the results, the 4‐week interval produced superior improvements in wrinkle scores. Because the 1927 nm laser stimulates collagen production and skin remodeling, a 4‐week interval may allow sufficient time for neocollagenesis to develop between sessions. A shorter 2‐week interval might not allow the full effects to manifest before the next treatment, which could limit the effectiveness. In addition, the cumulative benefits of collagen remodeling and skin rejuvenation are more likely to be visible with a longer gap between sessions. Nevertheless, the shorter 2‐week interval still achieved significant improvements in L* and pigmentation at the end of study visit compared to baseline, and did not increase the risk of adverse effects.

The primary concern in periorbital rejuvenation is safety, given the delicate nature of the periorbital area and its proximity to the eye, a vital organ. Pain levels during the initial laser treatment were assessed using a visual analogue scale, yielding an average rating of 5.5 (SD = 2.4), which underscores the heightened sensitivity of the periorbital region. Despite this discomfort, no severe adverse events related to eyesight, eye irritation, or scarring were observed. The predominant symptoms experienced following the treatment were erythema, swelling, itchiness, tenderness, and scaling, with most symptoms being of mild intensity. The duration of these discomforting symptoms typically lasts within 7 days. Overall, the safety profile of the 1927 nm laser treatment is deemed acceptable for periorbital management.

A limitation of the study is the small sample size, making it challenging to demonstrate statistical improvement in certain parameters. Although the improvement in skin quality was evident in the physician and subject GAIS, the ANTERA 3D texture score did not show improvement in the end‐of‐study visit. Another potential reason could be that the calculation of texture may not accurately reflect the improvements brought about by our treatment. The age difference between the two groups may, to some extent, have contributed to the better results observed in group B, as younger patients may have responded more effectively to the rejuvenation process than the older participants in group A. The increased net collagen accumulation in Group B, resulting from a longer treatment interval and follow‐up period since Visit 1, may have contributed to its superior efficacy in improving pores and wrinkles compared to Group A, as neocollagenesis is a time‐dependent process.

In summary, the 1927 nm diode laser demonstrates significant effectiveness for periorbital rejuvenation. The 4‐week interval treatment appears to be the optimal choice, showing superior improvements in wrinkles' score at the end‐of‐study visit (visit 4). Importantly, no severe adverse events were reported, with only mild, transient side effects such as tenderness, erythema, swelling, and scaling, most of which resolved within 7 days. Additionally, the noticeable improvements in skin brightness and the reduction of brown spots further highlight the unique benefits of the 1927 nm diode laser, setting it apart from other ablative and nonablative fractional lasers.

## Author Contributions

Chang‐Ming Huang and Yi‐Hua Liao contributed to the design and implementation of the research. Chang‐Ming Huang, Yi‐Shuan Sheen, and Yi‐Hua Liao contributed to the analysis of the results and the writing of the manuscript.

## Disclosure

This study was sponsored by BRIDGECON CO., LTD.

## Ethics Statement

The authors confirm adherence to the journal's ethical policies, as outlined in the author guidelines, and that approval has been obtained from the appropriate ethical review committee.

## Conflicts of Interest

The authors declare no conflicts of interest.

## Supporting information


Data S1.

## Data Availability

The data that support the findings of this study are available on request from the corresponding author. The data are not publicly available due to privacy or ethical restrictions.
