# Supplementary material for: Nonablative Fractional 1927‐nm Laser for Periorbital Rejuvenation: A Prospective, Double‐Arm, Open‐Label Trial
Source: J Cosmet Dermatol. 2025 Jun 19;24(6):e70274. doi: 10.1111/jocd.70274 (PMC12178097; doi:10.1111/jocd.70274)
Supplement: Supplementary file 1 — Data S1. [file JOCD-24-e70274-s001.docx]

**Supplementary Material**

**Supplementary Tables 1. Comparison of self-assessment questionnaire parameters across visits**

| group | parameter (median) | visit 1 | visit 2 | p value^†^ | visit 3 | p value^‡^ | visit 4 | p value^§^ |
| --- | --- | --- | --- | --- | --- | --- | --- | --- |
| A | brightness of skin | 4 | 3 | 0.0625 | 2 | 0.0313* | 2 | 0.0078* |
|  | fairness of skin | 3 | 3 | 0.75 | 2.5 | 0.125 | 2 | 0.0156* |
|  | translucency of skin | 4 | 3 | 0.125 | 2.5 | 0.0313* | 2 | 0.0078* |
|  | smoothness of skin to the touch | 3 | 3 | 1 | 2 | 0.25 | 2 | 0.0625 |
|  | softness of skin to the touch | 3 | 3 | 1 | 2 | 0.25 | 2 | 0.1094 |
|  | hydration level of skin | 3 | 3 | 1 | 2.5 | 0.25 | 2 | 0.1094 |
|  | radiance of skin | 3.5 | 3 | 0.125 | 2.5 | 0.0625 | 2 | 0.0078* |
|  | moisture retention of skin | 3 | 3 | 0.75 | 2.5 | 0.0625 | 2 | 0.0078* |
|  | evenness of skin tone | 3 | 3 | 1 | 3 | 0.375 | 2 | 0.0156* |
|  | elasticity of skin | 3 | 3 | 1 | 2 | 0.0625 | 2 | 0.0156* |
|  | overall health of skin | 3 | 3 | 0.25 | 2.5 | 0.0313* | 2 | 0.0039* |
|  | fullness of skin | 3 | 3 | 1 | 2.5 | 0.125 | 2 | 0.0156* |
|  | number of brown spots on skin | 4 | 3 | 0.125 | 3 | 0.0625 | 2 | 0.0078* |
|  | color of brown spots on skin | 4 | 3 | 0.125 | 3 | 0.0625 | 2 | 0.0078* |
|  | size of brown spots on skin | 4 | 3 | 0.125 | 3 | 0.0313* | 2 | 0.0078* |
|  | overall area of brown spots on skin | 3.5 | 3 | 0.25 | 3 | 0.0625 | 2 | 0.0078* |
|  | fine lines on skin | 3 | 3 | 1 | 3 | 1 | 2 | 0.1172 |
|  | youthfulness of skin appearance | 3 | 3 | 0.625 | 2 | 0.1875 | 2 | 0.0156* |
|  | refinement of skin texture | 3 | 3 | 0.25 | 3 | 0.25 | 2 | 0.0313* |
|  | tightness of skin | 3 | 3 | 0.75 | 3 | 0.25 | 2 | 0.0938 |
|  | feel like skin has been revitalized | 4 | 3 | 0.125 | 3 | 0.0313* | 2.5 | 0.0078* |
| B | brightness of skin | 4 | 2.5 | 0.0078* | 2 | 0.0039* | 2 | 0.0039* |
|  | fairness of skin | 4 | 2.5 | 0.0313* | 2 | 0.0078* | 2.5 | 0.0156* |
|  | translucency of skin | 4 | 3 | 0.0313* | 2 | 0.0078* | 2 | 0.0078* |
|  | smoothness of skin to the touch | 3 | 2.5 | 0.25 | 2 | 0.0547 | 2 | 0.0938 |
|  | softness of skin to the touch | 3 | 2 | 0.0625 | 2 | 0.0859 | 2 | 0.1563 |
|  | hydration level of skin | 4 | 3 | 0.1875 | 2 | 0.0156* | 2 | 0.0078* |
|  | radiance of skin | 4 | 3 | 0.0625 | 2 | 0.0039* | 2 | 0.0039* |
|  | moisture retention of skin | 4 | 3 | 0.0313* | 2 | 0.0078* | 3 | 0.0156* |
|  | evenness of skin tone | 4 | 3 | 0.25 | 2.5 | 0.0469* | 3 | 0.0313* |
|  | elasticity of skin | 3 | 3 | 0.3125 | 2 | 0.0313* | 2 | 0.125 |
|  | overall health of skin | 3 | 3 | 1 | 2 | 0.0313* | 2 | 0.2656 |
|  | fullness of skin | 3 | 3 | 0.9063 | 2 | 0.0586 | 2 | 0.0898 |
|  | number of brown spots on skin | 4 | 3 | 0.0313* | 2.5 | 0.0039* | 2.5 | 0.0039 |
|  | color of brown spots on skin | 4 | 3 | 0.0313* | 2.5 | 0.0039* | 3 | 0.0039* |
|  | size of brown spots on skin | 4 | 3 | 0.0313* | 2.5 | 0.0039* | 2.5 | 0.0039* |
|  | overall area of brown spots on skin | 4 | 3 | 0.0938 | 2 | 0.0078* | 2.5 | 0.0078* |
|  | fine lines on skin | 4 | 2.5 | 0.0039* | 2 | 0.0078* | 2 | 0.002* |
|  | youthfulness of skin appearance | 4 | 3 | 0.0156* | 2 | 0.0039* | 2 | 0.0039* |
|  | refinement of skin texture | 4 | 3 | 0.0313* | 2 | 0.0039* | 2 | 0.0039* |
|  | tightness of skin | 4 | 3 | 0.0313* | 2 | 0.0039* | 2 | 0.0078* |
|  | feel like skin has been revitalized | 4 | 3 | 0.1719 | 2 | 0.0078* | 2 | 0.0078* |

† The p-value obtained from the comparison between the visit 1 and visit 2.

‡ The p-value obtained from the comparison between the visit 1 and visit 3.

§ The p-value obtained from the comparison between the visit 1 and visit 4.

Data are shown as medians. *P ≤ 0.05. P value was calculated using a two-tailed Wilcoxon signed-rank test.

**Supplementary Tables 2. Adverse events**

| Group | Treatment number | Symptoms | None | Mild | Moderate | Severe |
| --- | --- | --- | --- | --- | --- | --- |
| A | 1^st^ treatment | Erythema | 2 (20%) | 6 (60%) | 2 (20%) | 0 (0%) |
|  |  | Swelling | 6 (60%) | 4 (40%) | 0 (0%) | 0 (0%) |
|  |  | Itchiness | 9 (90%) | 1 (10%) | 0 (0%) | 0 (0%) |
|  |  | Tenderness | 3 (30%) | 5 (50%) | 2 (20%) | 0 (0%) |
|  |  | Scaling | 7 (70%) | 2 (20%) | 1 (10%) | 0 (0%) |
|  | 2^nd^ treatment | Erythema | 1 (10%) | 5 (50%) | 4 (40%) | 0 (0%) |
|  |  | Swelling | 5 (50%) | 3 (30%) | 1 (10%) | 1 (10%) |
|  |  | Itchiness | 8 (80%) | 2 (20%) | 0 (0%) | 0 (0%) |
|  |  | Tenderness | 3 (30%) | 6 (60%) | 1 (10%) | 0 (0%) |
|  |  | Scaling | 4 (40%) | 5 (50%) | 1 (10%) | 0 (0%) |
|  | 3^rd^ treatment | Erythema | 1 (10%) | 7 (70%) | 2 (20%) | 0 (0%) |
|  |  | Swelling | 5 (50%) | 4 (40%) | 1 (10%) | 0 (0%) |
|  |  | Itchiness | 9 (90%) | 1 (10%) | 0 (0%) | 0 (0%) |
|  |  | Tenderness | 1 (10%) | 8 (80%) | 1 (10%) | 0 (0%) |
|  |  | Scaling | 5 (50%) | 4 (40%) | 1 (10%) | 0 (0%) |
| B | 1^st^ treatment | Erythema | 1 (10%) | 1 (10%) | 7 (70%) | 1 (10%) |
|  |  | Swelling | 1 (10%) | 5 (50%) | 2 (20%) | 2 (20%) |
|  |  | Itchiness | 6 (60%) | 3 (30%) | 1 (10%) | 0 (0%) |
|  |  | Tenderness | 1 (10%) | 3 (30%) | 4 (40%) | 2 (20%) |
|  |  | Scaling | 3 (30%) | 5 (50%) | 1 (10%) | 1 (10%) |
|  | 2^nd^ treatment | Erythema | 8 (80%) | 2 (20%) | 0 (0%) | 0 (0%) |
|  |  | Swelling | 1 (10%) | 9 (90%) | 0 (0%) | 0 (0%) |
|  |  | Itchiness | 5 (50%) | 4 (40%) | 1 (10%) | 0 (0%) |
|  |  | Tenderness | 1 (10%) | 8 (80%) | 1 (10%) | 0 (0%) |
|  |  | Scaling | 5 (50%) | 4 (40%) | 1 (10%) | 0 (0%) |
|  | 3^rd^ treatment | Erythema | 1 (10%) | 6 (60%) | 3 (30%) | 0 (0%) |
|  |  | Swelling | 2 (20%) | 6 (60%) | 2 (20%) | 0 (0%) |
|  |  | Itchiness | 5 (50%) | 3 (30%) | 2 (20%) | 0 (0%) |
|  |  | Tenderness | 2 (20%) | 6 (60%) | 1 (10%) | 1 (10%) |
|  |  | Scaling | 3 (30%) | 6 (60%) | 1 (10%) | 0 (0%) |

**Supplementary Table 3. Lasers for periorbital rejuvenation**

| Author | Year | Device | Clinical Outcomes | Side Effects |
| --- | --- | --- | --- | --- |
| Fournier et al^1^ | 2001 | NAFL 1540-nm Er:glass laser | Global mild improvement after 4 treatments | No side effects were reported by the subjects |
| Lupton et al^2^ | 2002 | Nonablative 1540-nm Er:glass laser | Slow, progressive clinical improvement of rhytides | Transient erythema and edema immediately |
| Tanzi et al^3^ | 2003 | Nonablative 1450-nm diode laser | Mild to moderate improvement in treated facial rhytides | Transient erythema, edema, and post-inflammatory hyperpigmentation (18%) |
| Kopera et al^4^ | 2004 | Nonablative 1450-nm diode laser | Mild to moderate improvement in all cases of wrinkles, skin texture and clinical appearance | Not mentioned |
| Alster et al^5^ | 2004 | Ablative CO_2_ laser | Significant improvement in dermatochalasis and rhytides | Erythema (2–4 weeks), hyperpigmentation (25%) |
| Manstein et al^6^ | 2004 | 1480-nm, 1535-nm, 1550-nm NAFL | Moderate improvement in wrinkles (in 34%) and texture (in 47%) | Mild edema (6–24 hours); no hyperpigmentation |
| Karabudak et al^7^ | 2008 | Q-switched Nd:YAG laser | 50% had statistically significant clinical improvement in rhytid severity | Pinpoint bleeding or erythema (3–5 days) |
| Karsai et al^8^ | 2010 | Fractional ablative CO_2_ and Er:YAG laser | Wrinkle depth and Fitzpatrick score reduced by  approximately 20% and 10%, with no difference between lasers | Erythema and swelling (6 days) |
| Kotlus^9^ | 2010 | Fractional ablative CO_2_ laser | 53% and 42% improvement in rhytides and skin redundancy | Erythema (21 days), hyperpigmentation (13%) |
| Jung et al^10^ | 2010 | NAFL 1550-nm and 1565-nm Er:glass | Improvement scores 2.25 ± 0.62 and 2.28 ± 0.59 respectively, with no difference between lasers | Erythema and edema (2-3 days), bronzing of the skin (30%), darkening of pre-existing pigmentary lesions (15%) |
| Lee et al^11^ | 2010 | 2,790-nm yttrium scandium gallium garnet (YSGG) laser | Clinical improvement by Fitzpatrick Wrinkle Classification System | Mild pain during treatment, mild erythema, and edema (5 days) |
| Ancona et al^12^ | 2010 | Fractional CO_2_ laser | Improvements in eyelid wrinkles, crow’s feet and skin laxity, 60% showed 26–50% improvement at three months | Redness and swelling (1 day), erythema and edema (3-4 days) |
| Tierney et al^13^ | 2011 | Fractional CO_2_ laser | Improvement in skin texture, skin laxity, and rhytids | Minor crusting and oozing (2-3 days), erythema and edema (7 days) |
| Leyden et al^14^ | 2012 | NAFL (1410 ± 5 nm) laser | Fitzpatrick Wrinkle Scale score improvement by one or more grades in 90% of subjects | Most prevalent side effect was transient posttreatment erythema |
| Wattanakrai et al^15^ | 2012 | fractional 1550-nm Yb/Er fiber laser or 2940-nm Er:YAG laser treatment | Wrinkle measurement 3 months after treatments –3.03 versus –3.09 respectively, with no difference between lasers | Erythema, edema, and burning sensation, less downtime correlated f the fractional laser treatment. |
| Chang et al^16^ | 2014 | long-pulsed Nd:YAG laser | Mean wrinkle score on the treated side decreased by 34.9% | Erythema in one subject (5%) lasting 2 weeks |
| Augustyniak et al^17^ | 2016 | NAFL 1410-nm laser | An improvement of the skin flexibility confirmed by reviscometer probe and photographical records. | Exfoliating crusts (100%), dry skin (54%), temporary erythema (54%), edema (15%), and petechial spots (39%) |
| Horovitz et al^18^ | 2021 | NAFL 1565-nm Er:glass laser | Fitzpatrick Wrinkle Scale scores improvement after 8 weeks | Minimal side effects and downtime |
| Salameh et al^19^ | 2021 | NAFL 1565-nm Er:glass laser | Fitzpatrick Wrinkle Scale score improvement of 1.7 | Mild burning and stinging (1–2 hours), pinpoint scabbing and crusting (1 day) |
| Badawi et al^20^ | 2022 | Non-ablative long-pulsed 2940 nm Er:YAG laser | Statistically and clinically significant improvement in the Fitzpatrick classification of the periorbital wrinkles | Erythema, edema (up to 12 hours), skin peeling (up to 5 days) |
| Sartori et al^21^ | 2022 | Fractional CO_2_ laser | Improvement of periorbital rhytidosis | Mild to moderate erythema and edema |
| Shenhav et al^22^ | 2023 | CO_2_ and 1570-nm hybrid laser | Objective improvement assessed by 4 independent physicians | Mild to moderate erythema, crusting, pruritus, edema, and hyperpigmentation |

**Reference of Supplementary Table 3**

1. Fournier N, Dahan S, Barneon G, et al. Nonablative remodeling: clinical, histologic, ultrasound imaging, and profilometric evaluation of a 1540 nm Er:glass laser. *Dermatol Surg.* 2001;27(9):799-806.

2. Lupton JR, Williams CM, Alster TS. Nonablative laser skin resurfacing using a 1540 nm erbium glass laser: a clinical and histologic analysis. *Dermatol Surg.* 2002;28(9):833-835.

3. Tanzi EL, Williams CM, Alster TS. Treatment of facial rhytides with a nonablative 1,450-nm diode laser: a controlled clinical and histologic study. *Dermatol Surg.* 2003;29(2):124-128.

4. Kopera D, Smolle J, Kaddu S, Kerl H. Nonablative laser treatment of wrinkles: meeting the objective? Assessment by 25 dermatologists. *Br J Dermatol.* 2004;150(5):936-939.

5. Alster TS, Bellew SG. Improvement of dermatochalasis and periorbital rhytides with a high-energy pulsed CO2 laser: a retrospective study. *Dermatol Surg.* 2004;30(4 Pt 1):483-487; discussion 487.

6. Manstein D, Herron GS, Sink RK, Tanner H, Anderson RR. Fractional photothermolysis: a new concept for cutaneous remodeling using microscopic patterns of thermal injury. *Lasers Surg Med.* 2004;34(5):426-438.

7. Karabudak O, Dogan B, Baloglu H. Histologic evidence of new collagen formation using a Q-switched Nd:YAG laser in periorbital rhytids. *J Dermatolog Treat.* 2008;19(5):300-304.

8. Karsai S, Czarnecka A, Jünger M, Raulin C. Ablative fractional lasers (CO(2) and Er:YAG): a randomized controlled double-blind split-face trial of the treatment of peri-orbital rhytides. *Lasers Surg Med.* 2010;42(2):160-167.

9. Kotlus BS. Dual-depth fractional carbon dioxide laser resurfacing for periocular rhytidosis. *Dermatol Surg.* 2010;36(5):623-628.

10. Jung JY, Cho SB, Chung HJ, Shin JU, Lee KH, Chung KY. Treatment of periorbital wrinkles with 1550- and 1565-nm Er:glass fractional photothermolysis lasers: a simultaneous split-face trial. *J Eur Acad Dermatol Venereol.* 2011;25(7):811-818.

11. Lee JW, Kim BJ, Kim MN, Lee CK. Treatment of periorbital wrinkles using a 2,790-nm yttrium scandium gallium garnet laser. *Dermatol Surg.* 2010;36(9):1382-1389.

12. Ancona D, Katz BE. A prospective study of the improvement in periorbital wrinkles and eyebrow elevation with a novel fractional CO2 laser--the fractional eyelift. *J Drugs Dermatol.* 2010;9(1):16-21.

13. Tierney EP, Hanke CW, Watkins L. Treatment of lower eyelid rhytids and laxity with ablative fractionated carbon-dioxide laser resurfacing: Case series and review of the literature. *J Am Acad Dermatol.* 2011;64(4):730-740.

14. Leyden J, Stephens TJ, Herndon JH, Jr. Multicenter clinical trial of a home-use nonablative fractional laser device for wrinkle reduction. *J Am Acad Dermatol.* 2012;67(5):975-984.

15. Wattanakrai P, Pootongkam S, Rojhirunsakool S. Periorbital rejuvenation with fractional 1,550-nm ytterbium/erbium fiber laser and variable square pulse 2,940-nm erbium:YAG laser in Asians: a comparison study. *Dermatol Surg.* 2012;38(4):610-622.

16. Chang SE, Choi M, Kim MS, Chung JY, Park YW, Lee JH. Long-pulsed Nd:YAG laser on periorbital wrinkles in Asian patients: randomized split face study. *J Dermatolog Treat.* 2014;25(4):283-286.

17. Augustyniak A, Rotsztejn H. Fractional non-ablative laser treatment at 1410 nm wavelength for periorbital wrinkles - reviscometrical and clinical evaluation. *J Cosmet Laser Ther.* 2016;18(5):275-279.

18. Horovitz T, Clementoni MT, Artzi O. Nonablative laser skin resurfacing for periorbital wrinkling-A case series of 16 patients. *J Cosmet Dermatol.* 2021;20(1):99-104.

19. Salameh F, Daniely D, Kauvar A, Carasso RL, Mehrabi JN, Artzi O. Treatment of periorbital wrinkles using thermo-mechanical fractional injury therapy versus fractional non-ablative 1565 nm laser: A comparative prospective, randomized, double-arm, controlled study. *Lasers Surg Med.* 2022;54(1):46-53.

20. Badawi A, Sobeih T, Jasmina V. Periocular rejuvenation using a unique non-ablative long-pulse 2940 nm Er:YAG laser. *Lasers Med Sci.* 2022;37(2):1111-1118.

21. de Filippi Sartori J, Osaki TH, Osaki MH, de Souza RB, Allemann N. "Split-Face" Evaluation of Collagen Changes Induced by Periorbital Fractional CO2 Laser Resurfacing. *Aesthet Surg J.* 2022;42(3):239-248.

22. Tsur Shenhav L, Shehade W, Muravnik G, Horovitz T, Artzi O. The Safety and Efficacy of a Dual CO 2 and 1570-nm Hybrid Laser for Periorbital Rejuvenation. *Dermatol Surg.* 2023;49(5):479-482.
